# Vibrio repeats (VXRs) play a role in horizontal dissemination of sedentary chromosomal integron-associated antibiotic resistance genes across Vibrio species

**DOI:** 10.1007/s10142-026-02068-8

**Published:** 2026-10-06

**Authors:** Sergio Mascarenhas Morgado, Erica Lourenço da Fonseca, Ana Carolina Paulo Vicente

**Affiliations:** Laboratório de Genética Molecular de Microrganismos, Instituto Oswaldo Cruz, FIOCRUZ, Avenida Brasil 4365, Manguinhos, Rio de Janeiro, Brazil

**Keywords:** *attC*, Recombination, Cassette, Evolution, Sedentary, Integrase

## Abstract

Sedentary chromosomal integrons (SCIs) are large integrons that harbor extensive arrays of gene cassettes and constitute major reservoirs of bacterial genetic diversity. In *Vibrio*, SCI cassettes are flanked by species-associated *attC* repeats, here referred to as *Vibrio* species repeats (VXRs). Although SCIs have been proposed as ancestral sources of antibiotic resistance genes (ARGs), the extent to which SCI-associated ARGs are exchanged among *Vibrio* species remains poorly understood. To investigate the dynamics of SCI-associated ARGs, we analyzed 37,812 genomes representing 181 *Vibrio* species. Using VXR mapping, comparative phylogenetics, and genome screening, we identified 8,679 putative ARG cassettes distributed across 8,517 genomes from 11 species. Despite the diversity of VXRs identified in SCIs, ARG cassettes were predominantly associated with a small subset of related VXRs shared among phylogenetically distant species, suggesting horizontal transfer of ARG-associated cassettes across the genus. Comparative analyses further suggested that members of the Cholera clade may act as potential sources of SCI-associated ARG dissemination. Five major ARG families were identified within SCIs, including *cat*B, *qnr*VC, *bla*_CARB_, *dfr*A, and *vat*F, some of which displayed phylogenetic patterns consistent with interspecies transfer. Functional characterization of a SCI-associated *qnr*VC4 cassette from *Vibrio mimicus* confirmed its ability to confer reduced susceptibility to ciprofloxacin in a heterologous host. Together, our findings indicate that a conserved subset of VXR backbones, mainly from the Cholera clade, plays a role in ARG dissemination across *Vibrio* species, highlighting SCIs as active reservoirs contributing to the emergence and spread of antimicrobial resistance.

**Supplementary Information:**

The online version contains supplementary material available at https://doi.org/10.1007/s10142-026-02068-8.

## Introduction

Integrons are genetic platforms specialized in the capture, excision, and expression of gene cassettes, flanked by species-specific direct repeats, through the action of a site-specific recombinase, the integrase IntI (Fluit and Schmitz 2004). While mobile integrons are well known for their crucial role in disseminating antimicrobial resistance genes (ARGs) among clinical pathogens, the vast majority of cassette diversity resides in sedentary chromosomal integrons (SCIs), also known as superintegrons (SIs), large and stable arrays of gene cassettes (up to 200 or more), which act as biobanks (Darracq et al. 2025). First characterized in *Vibrio cholerae*, SCIs represent one of the richest reservoirs of genetic innovation in bacteria and have been proposed as the ancestral source from which mobile integrons evolved (Rowe-Magnus and Mazel 2001). In *Vibrio*, the species-specific direct repeats that flank the gene cassettes are called *Vibrio* species repeats (VXRs).

A key feature of integron biology is the mobility of gene cassettes, which may undergo excision and reinsertion within an SCI or transfer between species. Experimental work has provided direct evidence that SCIs in *V. cholerae* are dynamic structures capable of cassette recruitment. Rowe-Magnus et al. (2002) showed real-time recruitment of a *V. cholerae* SCI-derived *cat* gene into a mobile class 1 resistance integron during antibiotic selection, after which it could be disseminated to clinically relevant pathogens via conjugation. SCI diversification can also occur under natural transformation conditions through competence-mediated exchange, including environmental settings, resulting in extensive cassette reshuffling and acquisition of novel genes (Debatisse et al. 2025; Righi et al. 2026). Comparative genomic studies also indicate that SCIs serve as sources of gene cassettes for mobile integrons. In a *V. cholerae* O1 strain, Fonseca et al. (2008) identified a *qnr*VC cassette, in a class 1 integron, flanked by *attC* sites highly similar to repeats found in the SCIs of *V. parahaemolyticus* and *V. cholerae*, suggesting movement of cassettes among species and between SCIs and mobile integrons. More broadly, reviews of SCI diversity in *Vibrio* highlight the frequent presence of cassettes associated with metabolism, stress resistance, and antimicrobial tolerance, many of which also occur in mobile integrons circulating among human pathogens (Rapa and Labbate 2013). Together, these observations support the view of SCIs as evolutionary hubs that generate, diversify, and disseminate gene cassettes, including ARGs, across bacterial populations.

Despite these advances, major questions remain unresolved. In particular, it is unclear to what extent ARG-containing cassettes within SCIs are endogenous to specific species or acquired horizontally from distantly related lineages. In addition, most knowledge of SCIs comes from *V. cholerae*. The genus *Vibrio* is exceptionally diverse, with numerous species harboring extensive SCIs of varying size, structure, and repeat composition (Gillings 2014). However, no large-scale study has evaluated the diversity and origin of SCI-associated ARGs across the genus or determined whether particular clades function as donors or recipients of cassette flow.

In this study, by combining VXR identification, genome mapping of VXR-flanked ARGs, and phylogenetics of repeats, cassettes, and host genomes, we aim to identify the SCI-associated ARGs and their distribution among *Vibrio* species.

## Methods

### Genome sampling, SCI detection and VXR extraction

A total of 37,812 *Vibrio* genomes, encompassing 181 species, were obtained from NCBI Genome dataset (Sept 2025). Among these, *V. cholerae* (14,034 genomes) and *V. parahaemolyticus* (14,351 genomes) were the most represented. To SCI detection and VXR extraction, *Vibrio* genomes exhibiting completeness > 90%, contamination < 6%, and ≤ 3 contigs were screened to select representative genomes with complete SCI, which resulted in the selection of 903 genomes. From this dataset, one random genome per species (*n* = 85 species) was retained for integron analysis.

The SCI of these 85 representative genomes was searched using Integron Finder v2.0.6 with –local_max parameter (Néron et al. 2022). Additionally, the putative SCIs had their integrase analyzed to confirm them as Int4, characteristic of SCIs. Integrase sequences were recovered and submitted to phylogenetic clustering, identifying 47 IntI4-like integrases. Thus, 47 of the 85 sequences were classified as SCIs. All *Vibrio attC*-associated repeats (VXRs) identified in these 47 SCIs were extracted, yielding a total of 4,259 VXR sequences. For each putative SCI, the corresponding VXR sequences were initially clustered using CD-HIT-EST v4.8.1 (Li and Godzik 2006) with the parameters “-c 0.8 -g 1 -G 1 -aS 0.7 -aL 0.7”, resulting in 531 representative VXR sequences. Since VXR prediction by Integron Finder could generate false-positive *attC* sites (such as degenerate VXRs or palindromic motifs), sequences shared across different species were considered to represent true integron repeats.

### ARG screening, VXR mapping, and secondary-structure analysis

ARGs were surveyed in the 37,812 *Vibrio* genomes using abricate tool (https://github.com/tseemann/abricate) with Comprehensive Antibiotic Resistance Database (CARD), considering a minimum threshold of ≥ 70% sequence identity and ≥ 70% coverage. Next, for each ARG, its nucleotide sequence plus 500 bp of each flanking region were extracted. The ARGs and their flanking regions were subjected to BLASTN using the 531 VXRs, predicted previously, as queries. Hits were retained if: coverage > 70%, identity > 70%, alignment length > 95 bp, and bordering both sides of the ARG. The positions of these hits were then compared with the annotated ARG coordinates to classify each hit as upstream or downstream of the ARG. When multiple VXR references were detected on both sides of the same ARG, the two flanking hits corresponding to each VXR reference were evaluated jointly using a combined bitscore, calculated as the sum of the upstream and downstream bitscores. For each cassette, the VXR reference with the highest combined bitscore was selected, and the corresponding VXR nucleotide sequences were subsequently extracted directly from the ARG-containing sequence using the BLAST subject coordinates.

Representative VXR sequences were analyzed using mLocARNA v1.9.2 (https://github.com/s-will/LocARNA) to generate multiple sequence-structure alignments based on simultaneous sequence alignment and secondary-structure prediction. Secondary structures were predicted using the Mathews 2004 DNA thermodynamic parameters (dna_mathews2004.par; ViennaRNA Package) through the “--rnafold-parameter option”. The resulting consensus structures were examined to assess the conservation of secondary-structure features.

### Phylogenetic analyses

Representative genomes were randomly selected from each species containing VXR-associated ARGs, together with genomes harboring the same VXRs but lacking ARGs, and used for phylogenomic inference based on a concatenated alignment of single-copy core proteins identified using Anvi’o v9 (Eren et al. 2015). Maximum-likelihood phylogenetic trees (including core genome, gene, and VXR trees) were inferred with IQ-TREE v2.4.0 (Minh et al. 2020) considering 1,000 ultrafast bootstrap replicates, and MFP parameter. Prior to phylogenetic inference, the sequences comprising each gene dataset and the VXR dataset were independently clustered using CD-HIT-EST v4.8.1 (Li and Godzik 2006). Representative sequences from each resulting cluster were then selected and used for phylogenetic reconstruction. Gene sequences were clustered using the parameters “-g 1 -G 1 -c 0.9 -aS 0.7 -aL 0.7”, while VXR sequences were clustered using “-g 1 -G 1 -c 0.95 -aS 0.9 -aL 0.9”.

### Functional analysis of a SCI-associated ARG in heterologous system

To assess the functionality of ARGs of SCIs, we examined a *V. mimicus* strain (VM343) isolated from the environment (water) in 1990 in Japan (Morgado et al. 2024). Genomic sequencing of the strain was performed using ONT technology with a PromethION P2 Solo and a PromethION Flow Cell (FLO-PRO114M), alongside the Native Barcoding Kit 96 V14 (SQK-NBD114.96). The MinKNOW program was used for sequencing management, including real-time monitoring, and basecalling was performed using the maximum accuracy model v5.0.0–400 bps. The genome was assembled using Flye v2.9.6 with the nano-hq mode, and deposited in the Genbank (JAZHPO000000000.2).

Specific primers were used to amplify the complete *qnr*VC gene (5’- GAACGTCAATTTGGCACCT − 3’ and 5’- CTGTCAGGATTGCGACTCAT-3’) from strain VM343. The PCR product was cloned into the pGEM T-Easy Cloning Vector System (Promega) and used to transform *Escherichia coli* DH5α. The competent cells were plated onto LB containing ampicillin 100 µg/mL. After confirming the insert orientation relative to the vector promoter, the ciprofloxacin MICs for the transformants were determined using the Etest method (bioMérieux) on Mueller-Hinton agar. *E. coli* DH5α was used as a control, exhibiting a ciprofloxacin MIC of 0.05 µg/mL.

## Results

### *Vibrio* species carrying ARGs in the SCI

**Fig. 1:**
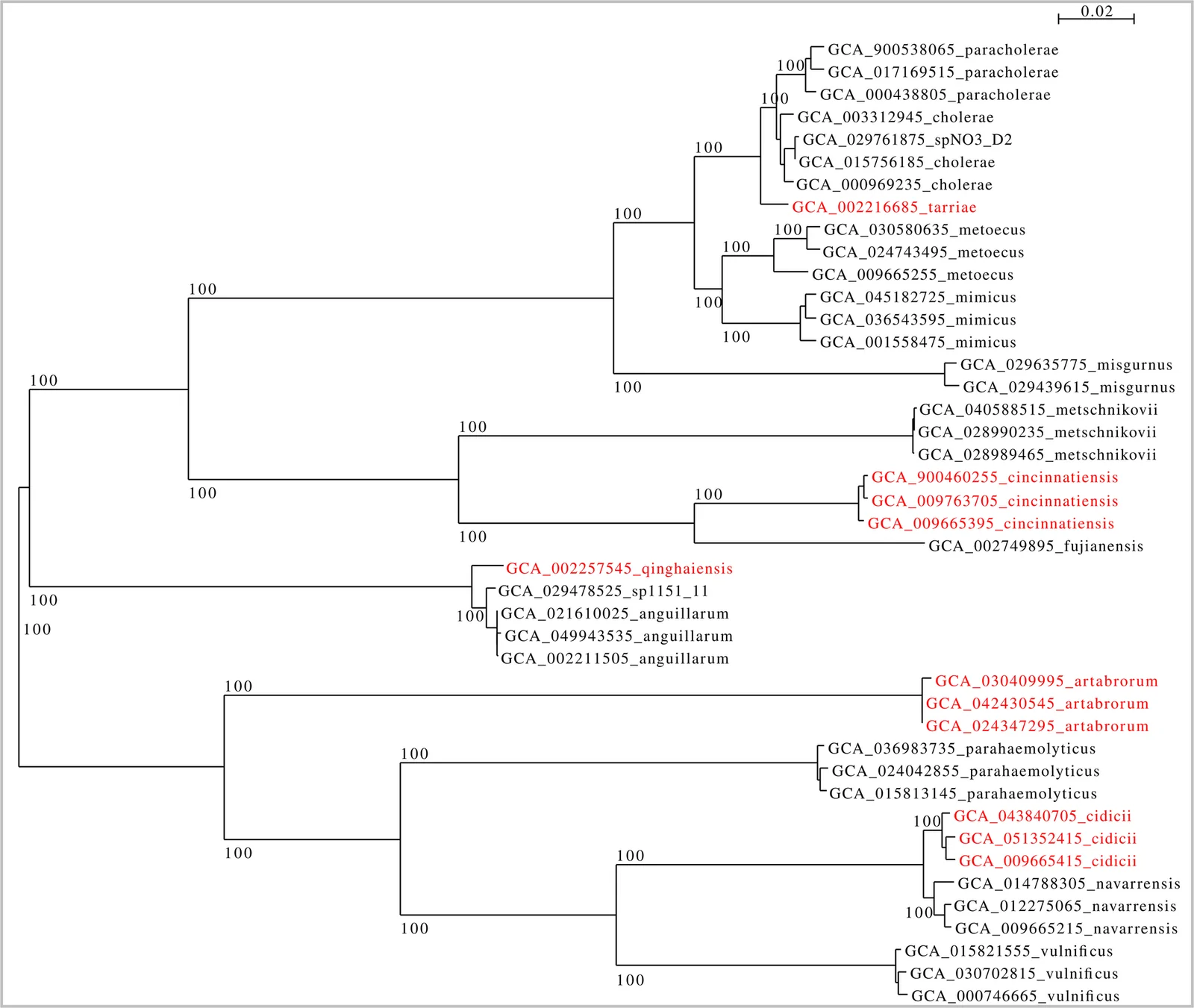
Phylogenomic relationships among representative *Vibrio* species. The maximum-likelihood phylogeny was reconstructed from a concatenated alignment of 744 single-copy core proteins identified using Anvi’o. The tree includes representative genomes from species harboring ARG-associated VXRs, as well as genomes from species carrying these VXRs in the absence of detected ARG-associated cassettes (highlighted in red)

Screening of 37,812 *Vibrio* genomes (181 species) for antibiotic resistance gene cassettes associated with SCIs (ARG/SCI) revealed putative 8,679 cassette sequences from 8,517 genomes (~ 22%). These sequences were distributed in the 11 species, in addition to two sp. genomes (Supplementary Material 1, Table S1): *Vibrio fujianensis* (1/1), *Vibrio misgurnus* (1/2), *Vibrio navarrensis* (1/46), *Vibrio vulnificus* (4/2,806), *Vibrio metoecus* (6/41), *Vibrio metschnikovii* (8/191), *Vibrio paracholerae* (19/63), *Vibrio mimicus* (20/116), *Vibrio parahaemolyticus* (22/14,351), *Vibrio anguillarum* (58/301), and *Vibrio cholerae* (8,375/14,034). Indeed, further analysis confirmed the presence of IntI4-like integrase in all of these genomes (data not shown). Although *V. cholerae* and *V. parahaemolyticus* genomes are overrepresented, a distinction is visible among species concerning ARG/SCIs. Despite both species having thousands of sequenced genomes, *V. parahaemolyticus* harbored only a minor fraction of genomes containing ARG/SCIs. Of these 8,517 genomes presenting ARG/SCIs, 4,354 (~ 51%) presented metadata associated with their source, where 3,669, 523, and 162 were from humans (3,664 from *V. cholerae*), environments, and animals, respectively (Supplementary Material 1, Table S1). ARGs have been presented within *Vibrio* SCIs for several decades, including in a *V. paracholerae* genome from 1916 (pre-antibiotic era) (Supplementary Material 1, Table S1). Most genomes carried only one ARG cassette in SCI, but 158 genomes carried two or three, almost exclusively from *V. cholerae* (149 genomes). A core genome phylogeny of the *Vibrio* species containing ARGs associated with VXRs, as well as genomes presenting these VXRs but lacking ARGs, revealed that ARG/SCI-carrying genomes were distributed across distinct *Vibrio* clades, with most clustering closely with *V. cholerae* (Fig. 1).

### Diversity of ARGs carried by SCIs

The 8,679 ARG/SCIs comprised five gene families: *dfrA*, *catB*, *qnrVC*, *bla*_CARB_ and *vatF*, though allelic diversity was observed (Table 1). These genes were distributed unevenly in the species, with *vat*F only in two; *dfr*A, *cat*B and *bla*_CARB_ in three; and *qnr*VC in nine species. In particular, *cat*B9 had an overwhelming dominance (7,694/8,679; ~88%), however, most were from *V. cholerae* (*n* = 7,691). Phylogenetic analyses of representative sequences of these genes, clustered at a 90% nucleotide identity threshold, showed that some *catB9* and *qnrVC* sequences from different *Vibrio* species clustered together, providing evidence consistent with their horizontal transfer (Supplementary Material 2, Fig. S1A-E). Indeed, some species harboring these genes were recovered from diverse sources, including clinical, animal, and environmental settings (Supplementary Material 1, Table S1).

**Table 1:** VXR-associated ARG distribution and diversity in *Vibrio* SCIs

ARG family	Total	Species (# genomes)
*catB9*	7,694	*V. navarrensis* (1), *V. parahaemolyticus* (1), *V.* sp. NO3-D2 (1), *V. cholerae* (7,691)
*qnrVC4*	452	*V. misgurnus* (1), *V. vulnificus* (1), *V. paracholerae* (2), *V. mimicus* (19), *V. cholerae* (429)
*blaCARB-7*	310	*V. metoecus* (4), *V. paracholerae* (4), *V. cholerae* (302)
*qnrVC7*	63	*V. mimicus* (1), *V. metoecus* (3), *V. vulnificus* (3), *V. cholerae* (56)
*qnrVC6*	71	*V. fujianensis* (1), *V.* sp. 1151_11 (1), *V. parahaemolyticus* (11), *V. anguillarum* (58)
*qnrVC5*	28	*V. cholerae* (28)
*blaCARB-9*	15	*V. paracholerae* (15)
*vatF*	6	*V. metschnikovii* (1), *V. paracholerae* (5)
*dfrA31*	14	*V. parahaemolyticus* (1), *V. cholerae* (13)
*dfrA6*	26	*V. cholerae* (9), *V. parahaemolyticus* (9), *V. metschnikovii* (8)
Total	8,679	

### Dominance of small sets of highly conserved VXRs

Of the 531 representative VXR sequences used for screening, 63 showed hits to ARG-containing sequences, but only eight were highly recurrent. These eight most prevalent sequences are 129–131 bp in length, share ≥ 90% nucleotide identity with each other, and matched over 8,300 of the 8,679 ARG-associated cassettes (Supplementary Material 3, Table S2, set 1). However, although highly prevalent among SCI-associated ARG cassettes, these VXRs were also associated with other cassette types.

Restricting the analysis to ARG cassettes from non–*V. cholerae* genomes (149 cassettes from 140 genomes) revealed that all eight aforementioned VXRs (Supplementary Material 3, Table S2, set 1) were still represented. Furthermore, an additional, distinct set of VXR sequences associated with ARGs was detected exclusively within these non–*V. cholerae* genomes (Supplementary Material 3, Table S2, set 2).

To determine whether specific VXR sequences are associated with ARGs, we reconstructed a phylogeny using representative VXR sequences from 8,679 ARG-associated cassettes (Supplementary Material 3, Table S3) and the initially described representative VXRs (Supplementary Material 3, Table S2, sets 1 and 2) (Supplementary Material 2, Fig. S2). The resulting tree revealed multiple clades: the most prevalent VXR sequences (Supplementary Material 3, Table S2, set 1) clustered within distinct subclades (Supplementary Material 2, Fig. S2, red background), whereas VXRs derived from non-*V. cholerae* genomes (Supplementary Material 3, Table S2, set 2) formed a separate branch (Supplementary Material 2, Fig. S2, blue background). Interestingly, a separate clade was predominantly associated with *dfrA*-containing cassettes (Supplementary Material 2, Fig. S2, green background), whereas the remaining ARGs clustered primarily within the clade formed by the eight most prevalent VXR sequences (Supplementary Material 2, Fig. S2, red background). These patterns suggest that specific VXR backbones may be linked to particular ARG-containing cassettes across diverse *Vibrio* species.

Secondary structure analyses of selected VXR clades, including the prevalent ones (Supplementary Material 3, Table S2, sets 1 and 2) and a clade associated with *dfrA*-containing cassettes (Supplementary Material 2, Fig. S2, green background), revealed highly conserved structures. Furthermore, these structures preserved functional signatures essential for integrase recognition and site-specific recombination (Supplementary Material 2, Fig. S3), consistent with previously described VXR/*attC* secondary structures (Bouvier et al. 2009).

Next, we evaluated the SCI of specific genomes from which each prevalent VXR sequence originated (Supplementary Material 3, Table S2, sets 1 and 2) to determine whether that repeat type was also highly represented within its native SCI. For example, does the GCA_000969235.1_V.cholerae_seq8 sequence predominate among the SCI repeats in the GCA_000969235.1 genome? This analysis enabled us to infer whether a prevalent VXR (Supplementary Material 3, Table S2, sets 1 and 2) was likely endogenous to its source SCI or instead represented a repeat associated with cassettes horizontally acquired. These results showed that within the Cholera clade (*V. cholerae*, *V. metoecus*, *V. paracholerae*, and *V. tarriae*), the predominant repeats of each analyzed genome matched the same VXR type found to be prevalent in the entire dataset (Supplementary Material 3, Table S2, set 1). The only partial exception was *V. mimicus* (GCA_001558475.2), in which the VXR GCA_001558475.2_V.mimicus_seq11 was not the dominant repeat but the second most abundant within its SCI. Indeed, *V. mimicus* is a basal lineage of the Cholera clade (Fig. 1). In contrast, the prevalent VXRs from *V. anguillarum*, *V. cidicii*, *V. cincinnatiensis*, and *V. qinghaiensis* (Supplementary Material 3, Table S2, set 1) were not dominant in their respective native SCIs. Furthermore, among the VXRs derived from non-*V. cholerae* genomes (Supplementary Material 3, Table S2, set 2), only those from *V. anguillarum* (seq5) and *V. qinghaiensis* (seq6) were the most prevalent within their respective SCIs. Taken together, these results indicate that, for this small set of genomes analyzed, ARG cassettes linked to the prevalent VXRs are likely endogenous to members of the Cholera clade, whereas they appear to be exogenous acquisitions in some other *Vibrio* species.

### Functional analyses of a SCI-associated ARG

To assess the functionality of an ARG cassette within SCI, we examined a *V. mimicus* strain VM343. Using long-read genome sequencing, we confirmed that the *qnrVC4* gene is located within the VM343 SCI, positioned 135 cassette genes downstream of *int*I4 (approximately 103 kb apart). The VXR sequence flanking this gene exhibited high similarity to the 11 VXRs inferred in this study (Table 1). This *qnr*VC allele was cloned and expressed in a heterologous system to evaluate its functionality. The resulting transformants displayed a ciprofloxacin MIC of 0.38 µg/mL, representing a nearly 8-fold increase compared to the control strain (0.05 µg/mL), a resistance increase typical of *qnrVC* alleles (Miranda et al. 2022), thereby confirming the expression and activity of the cassette.

## Discussion

SCIs have long been recognized as major reservoirs of gene cassettes, including ARGs and antiphage defense systems (Rowe-Magnus and Mazel 2001; Darracq et al. 2025; Righi et al. 2026). However, their contribution to the evolution and dissemination of ARGs across *Vibrio* species remains poorly understood. By combining VXR profiling, large-scale BLAST-based mapping of cassette boundaries, and phylogenetic analyses across thousands of *Vibrio* genomes, our study provides an overview of SCI-associated ARGs within the genus. Although a diverse array of VXRs was identified, ARG cassettes were predominantly linked to a small subset of highly conserved VXR types enriched among Cholera clade members, suggesting that specific VXR backbones are key mediators of SCI cassette mobilization.

SCI *attC* sites from *V.cholerae* are known to be highly conserved (Mazel 2006), a feature that would be expected to restrict cassette recruitment between distinct SCIs (Rowe-Magnus and Mazel 2001; Mazel 2006; Debatisse et al. 2025). However, our analysis revealed highly similar ARG-associated VXR sequences shared among phylogenetically distant *Vibrio* species. These patterns are consistent with interspecies cassette exchange, suggesting that these sites may retain structural compatibility with multiple IntI integrases, thereby facilitating interspecies cassette dissemination among SCIs. Indeed, Vit et al. (2021) demonstrated that integrase recognition of *attC* sites depends primarily on secondary structure rather than primary nucleotide sequence.

*Vibrio* species carrying ARGs within their SCIs were identified across One Health axes, even in contexts apparently lacking antibiotic selective pressure, such as the presence of ARGs in isolates from the pre-antibiotic era. These observations reinforce the role of SCIs as long-term reservoirs of adaptive genes (Gillings 2014) that further could be recruited into mobile integrons (Mazel 2006). Indeed, some ARG cassettes identified here have also been reported in class 1 integrons, often as distinct allelic variants. Examples include *bla*_CARB_ in *Pseudomonas aeruginosa* (AY913771) and *Salmonella enterica* (AY123251); *cat*B in *Acinetobacter berezinae* (EF125010) and *Klebsiella pneumoniae* (FJ594766); *dfr*A in Bacteroides fragilis (KF188200) and *K. pneumoniae* (CP003225); *qnr*VC in *Aeromonas punctata* (GQ891757) and *V. cholerae* (EU436855).

By comparing the most prevalent ARG-associated VXRs against the VXR repertoire of representative SCIs, we were able to infer potential directional patterns of ARG dissemination among species. Within the Cholera clade, each of these highly prevalent VXRs (Supplementary Material 3, Table S2, set 1) was also the most abundant repeat within the SCI of its corresponding genome. Conversely, in other *Vibrio* species, we sporadically identified ARG cassettes carrying these prevalent VXRs, suggesting horizontal acquisition. Together, these findings support the hypothesis that members of the Cholera clade may act as major sources of SCI-associated ARG dissemination across the genus. This expands upon the in vitro results of Righi et al. (2026), who showed that cross-species transfer of SCI cassettes can occur naturally from *Vibrio furnissii* and *V. parahaemolyticus* to *V. cholerae*.

This study has several limitations that should be considered. The identification of VXRs from ARG cassettes relied primarily on computational prediction and sequence similarity. VXR diversity was initially characterized from one representative genome per species, which may underestimate intraspecific variation of VXR. Furthermore, the skewed representation of *Vibrio* species in genomic databases, particularly the overrepresentation of human-clinical *V. cholerae* isolates, could confound estimates of VXR and ARG prevalence across the genus. Consequently, broader experimental validation coupled with structural analyses of *attC* sites will be essential to ascertain whether the conserved VXR patterns identified in our study represent the dominant recombination backbones driving ARG dissemination, or merely a subset of a wider array of functional *attC* sites. 

## Supplementary Information

Below is the link to the electronic supplementary material.

Supplementary Material 1

Supplementary Material 2

Supplementary Material 3

## Data Availability

No datasets were generated or analysed during the current study.

## References

[CR1] Bouvier M, Ducos-Galand M, Loot C et al (2009) Structural features of single-stranded integron cassette attC sites and their role in strand selection. PLoS Genet 5(9):e1000632. 10.1371/journal.pgen.1000632 PMC2727003 19730680

[CR2] Darracq B, Littner E, Brunie M et al (2025) Sedentary chromosomal integrons as biobanks of bacterial antiphage defense systems. Science 388(6747):eads0768. 10.1126/science.ads0768 40339013

[CR3] Debatisse K, Brunie M, Darracq B et al (2025) Bacterial natural transformation drives cassette shuffling and simplifies recombination in chromosomal integrons. Nucleic Acids Res 53(21):gkaf1162. 10.1093/nar/gkaf1162 PMC12614218 41242524

[CR4] Eren AM, Esen ÖC, Quince C et al (2015) Anvi’o: an advanced analysis and visualization platform for ‘omics data. PeerJ 3:e1319. 10.7717/peerj.1319 PMC4614810 26500826

[CR5] Fluit AC, Schmitz FJ (2004) Resistance integrons and super-integrons. Clin Microbiol Infect 10(4):272–288. 10.1111/j.1198-743X.2004.00858.x 15059115

[CR6] Fonseca EL, Dos Santos Freitas F, Vieira VV, Vicente AC (2008) New qnr gene cassettes associated with superintegron repeats in Vibrio cholerae O1. Emerg Infect Dis 14(7):1129–1131. 10.3201/eid1407.080132 PMC2600354 18598639

[CR7] Gillings MR (2014) Integrons: past, present, and future. Microbiol Mol Biol Rev 78(2):257–277. 10.1128/MMBR.00056-13 PMC4054258 24847022

[CR8] Li W, Godzik A (2006) Cd-hit: a fast program for clustering and comparing large sets of protein or nucleotide sequences. Bioinformatics 22(13):1658–1659. 10.1093/bioinformatics/btl158 16731699

[CR9] Mazel D (2006) Integrons: agents of bacterial evolution. Nat Rev Microbiol 4(8):608–620. 10.1038/nrmicro1462 16845431

[CR10] Minh BQ, Schmidt HA, Chernomor O et al (2020) IQ-TREE 2: new models and efficient methods for phylogenetic inference in the genomic era. Mol Biol Evol 37(5):1530–1534. 10.1093/molbev/msaa015 PMC7182206 32011700

[CR11] Miranda CD, Concha C, Godoy FA, Lee MR (2022) Aquatic environments as hotspots of transferable low-level quinolone resistance and their potential contribution to high-level quinolone resistance. Antibiot (Basel) 11(11):1487. 10.3390/antibiotics11111487 PMC9687057 36358142

[CR12] Morgado SM, Dos Santos Freitas F, Lourenço da Fonseca E, Vicente ACP (2024) Vibrio mimicus Lineage Carrying Cholera Toxin and Vibrio Pathogenicity Island, United States and China. Emerg Infect Dis 30(8):1729–1732. 10.3201/eid3008.240252 PMC11286048 39043427

[CR13] Néron B, Littner E, Haudiquet M, Perrin A, Cury J, Rocha EPC (2022) IntegronFinder 2.0: Identification and Analysis of Integrons across Bacteria, with a Focus on Antibiotic Resistance in Klebsiella. Microorganisms 10(4):700. 10.3390/microorganisms10040700 PMC9024848 35456751

[CR14] Rapa RA, Labbate M (2013) The function of integron-associated gene cassettes in Vibrio species: the tip of the iceberg. Front Microbiol 4:385. 10.3389/fmicb.2013.00385 PMC3856429 24367362

[CR15] Righi L, Stutzmann S, Bader L, Lemopoulos A, Blokesch M (2026) Competence-mediated DNA uptake diversifies Vibrio cholerae sedentary chromosomal integrons. Science 392(6794):194–201. 10.1126/science.aed0645 41955379

[CR17] Rowe-Magnus DA, Mazel D (2001) Integrons: natural tools for bacterial genome evolution. Curr Opin Microbiol 4(5):565–569. 10.1016/s1369-5274(00)00252-6 11587934

[CR16] Rowe-Magnus DA, Guerout AM, Mazel D (2002) Bacterial resistance evolution by recruitment of super-integron gene cassettes. Mol Microbiol 43(6):1657–1669. 10.1046/j.1365-2958.2002.02861.x 11952913

[CR18] Vit C, Richard E, Fournes F et al (2021) Cassette recruitment in the chromosomal Integron of Vibrio cholerae. Nucleic Acids Res 49(10):5654–5670. 10.1093/nar/gkab412 PMC8191803 34048565

