# Supplementary material for: Vibrio repeats (VXRs) play a role in horizontal dissemination of sedentary chromosomal integron-associated antibiotic resistance genes across Vibrio species
Source: Funct Integr Genomics. 2026 Oct 6;26(1):284. doi: 10.1007/s10142-026-02068-8 (PMC13638679; doi:10.1007/s10142-026-02068-8)
Supplement: Supplementary file 1 [file 10142_2026_2068_MOESM2_ESM.pdf]

Supplementary material 2

Vibrio Repeats (VXRs) play a role in horizontal dissemination of sedentary chromosomal integron-associated antibiotic resistance genes across Vibrio species

Functional & Integrative Genomics

Sergio Mascarenhas Morgado*, Erica Lourenço da Fonseca, Ana Carolina Paulo Vicente

Laboratório de Genética Molecular de Microrganismos, Instituto Oswaldo Cruz, FIOCRUZ. Avenida Brasil 4365, Manguinhos, Rio de Janeiro, Brazil

*Corresponding author. E-mail: sergio.morgado@ioc.fiocruz.br (S. Morgado)

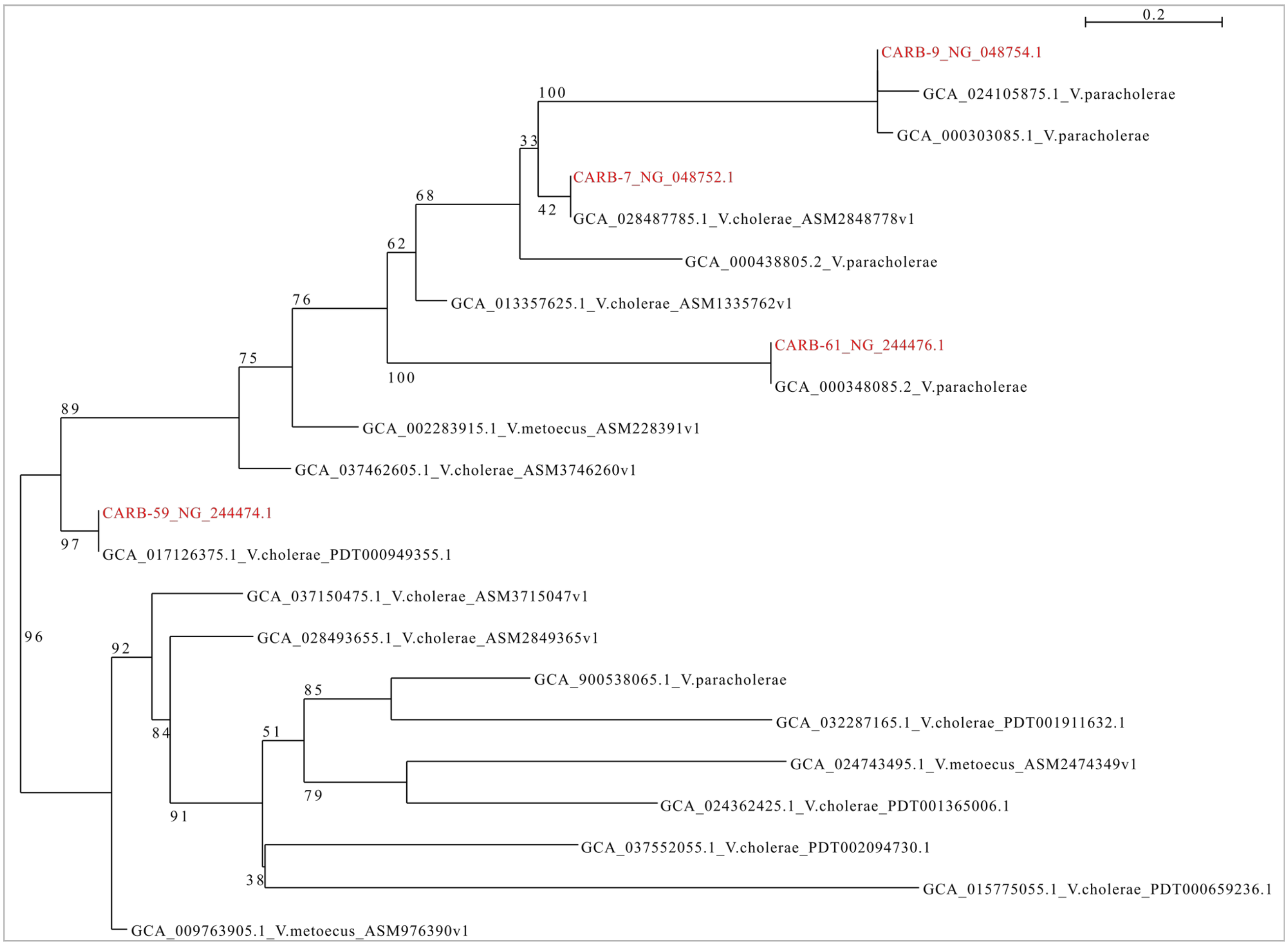

Figure S1A. Maximum-likelihood phylogenetic tree of SCI-associated *bla*CARB. The reference sequences are colored in red.

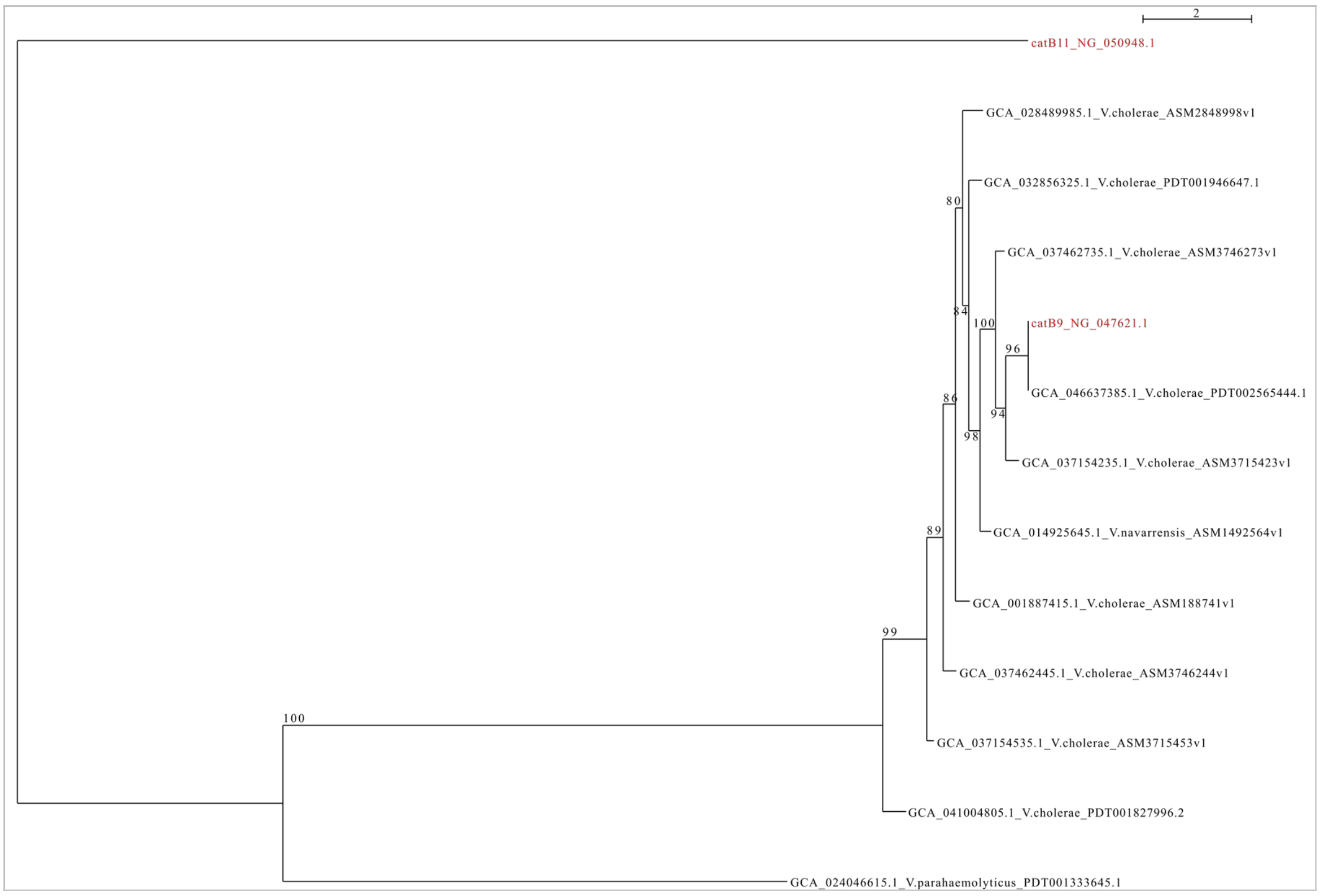

Figure S1B. Maximum-likelihood phylogenetic tree of SCI-associated *cat*. The reference sequences are colored in red.

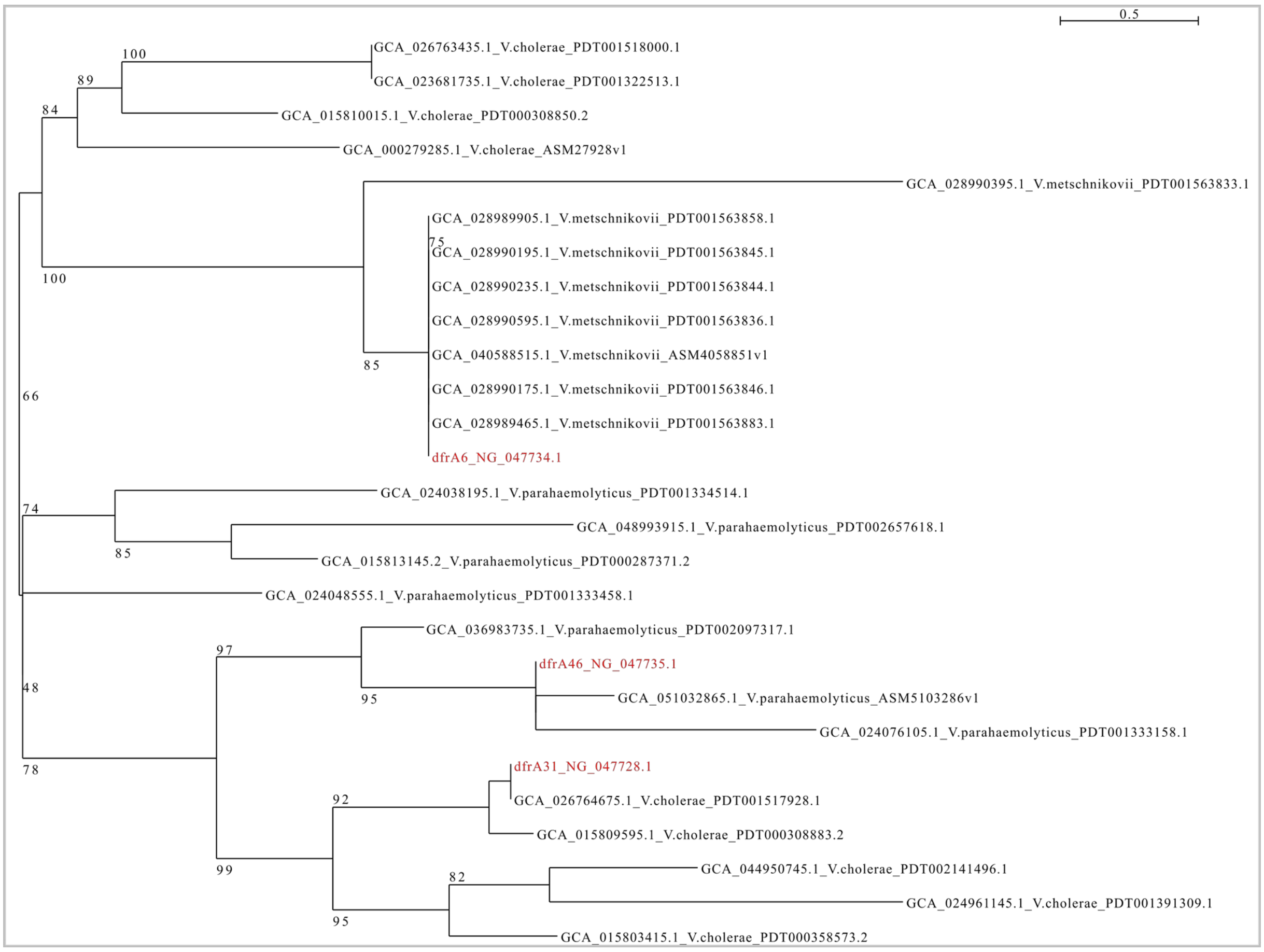

Figure S1C. Maximum-likelihood phylogenetic tree of SCI-associated *dfr*A. The reference sequences are colored in red.

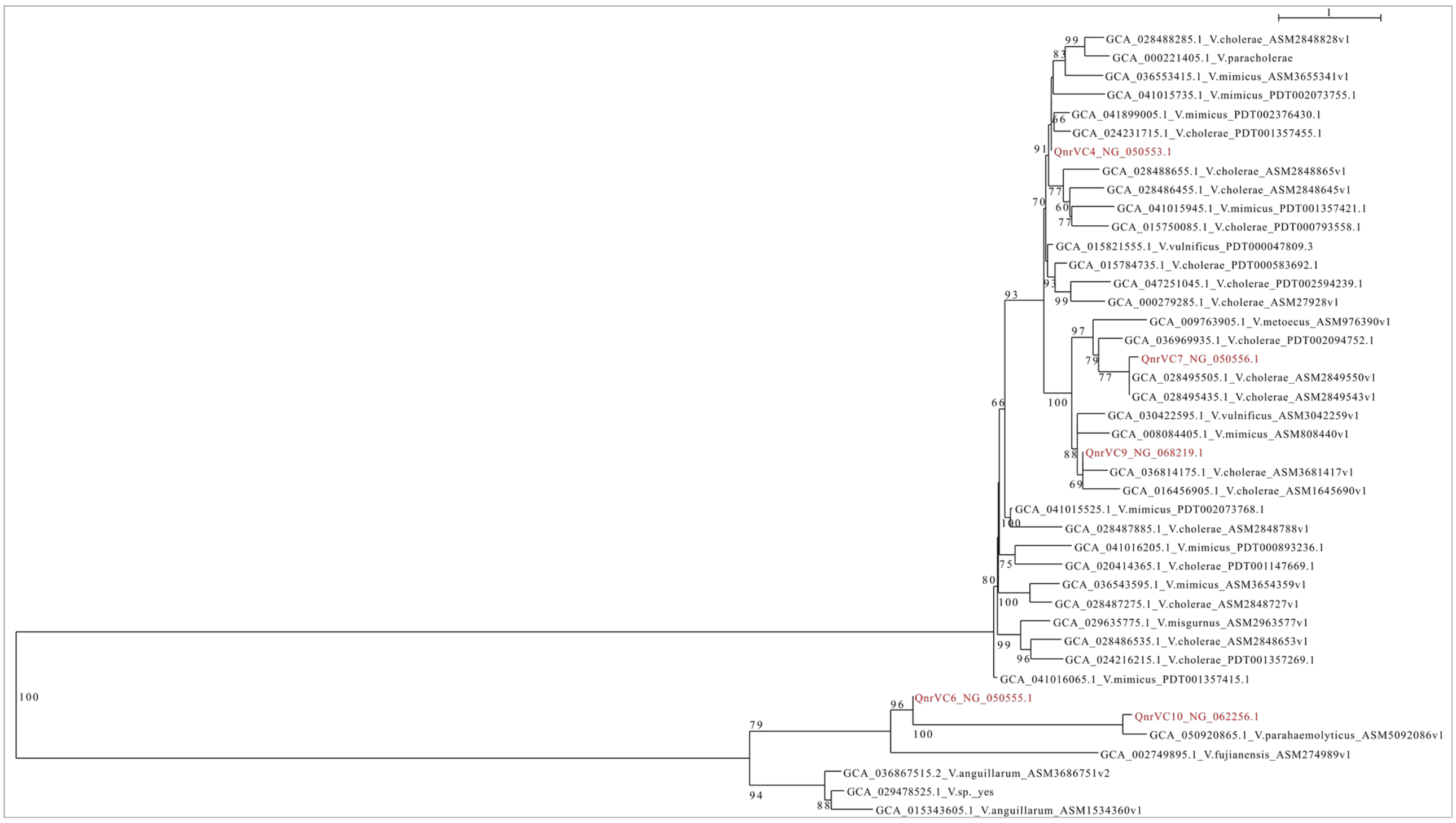

Figure S1D. Maximum-likelihood phylogenetic tree of SCI-associated *qnr*. The reference sequences are colored in red.

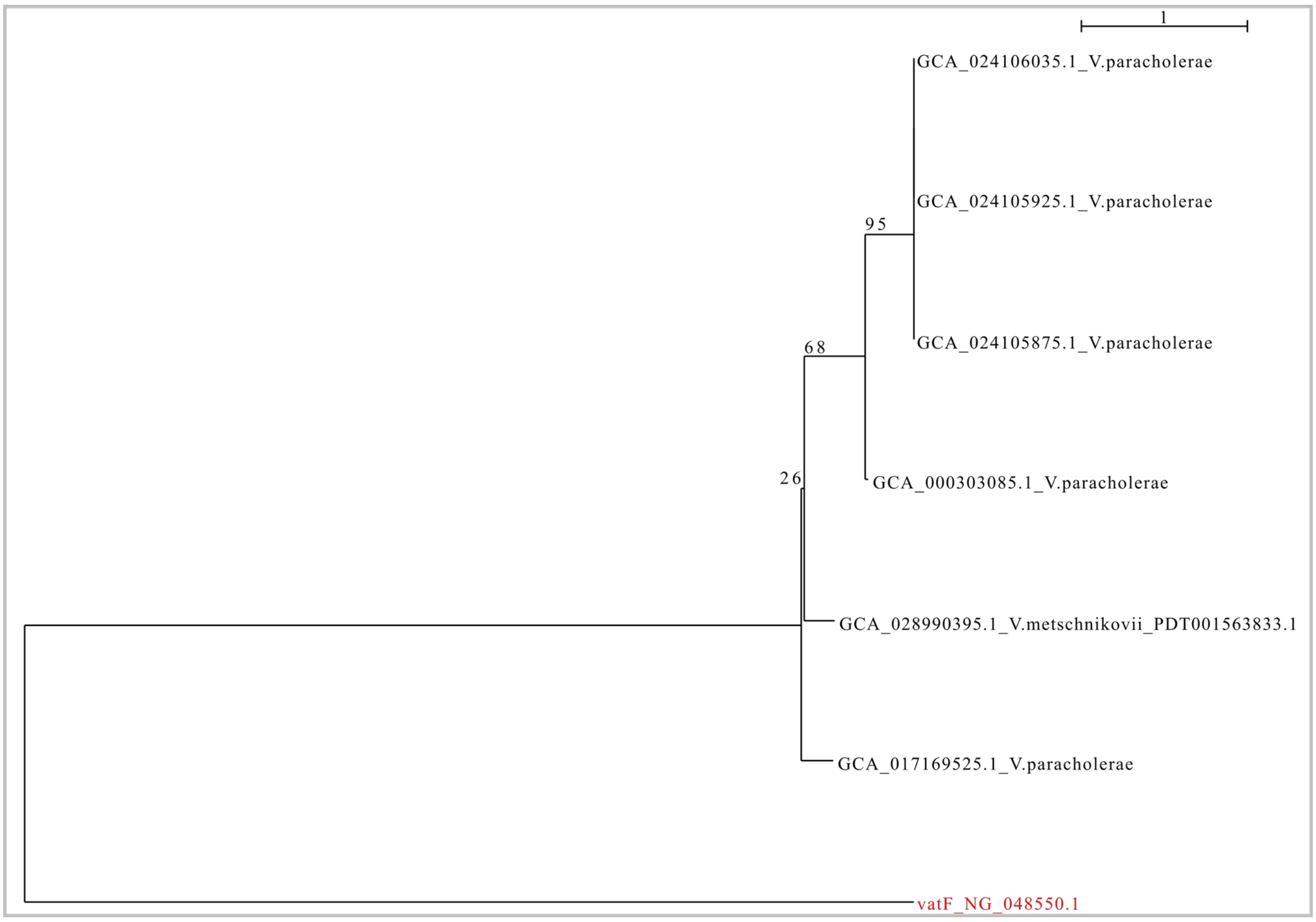

Figure S1E. Maximum-likelihood phylogenetic tree of SCI-associated *vatF*. The reference sequence is colored in red.

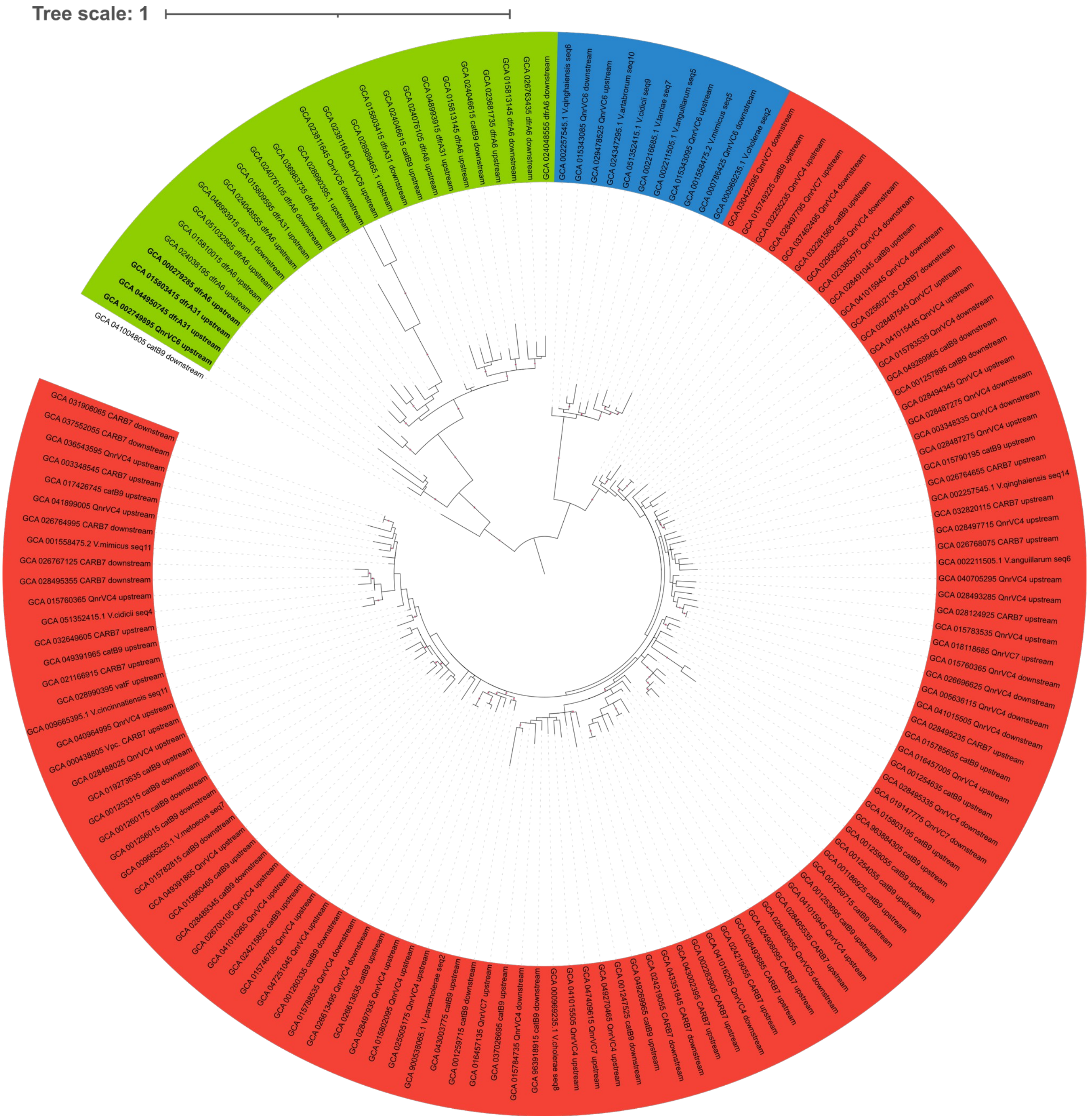

Figure S2. Maximum-likelihood phylogram of the most prevalent VXR sequences associated with ARG-containing cassettes. Red clades indicate the placement of the most prevalent VXR reference sequences in the dataset (Supplementary Material 3, Table S2, set 1), whereas blue clades comprise ARG-associated VXRs detected in non-*V. cholerae* genomes (Supplementary Material 3, Table S2, set 2). ARG families were not evenly distributed across the VXR phylogeny, with *qnrVC*, *catB9*, *vatF*, and *blaCARB* predominantly associated with the red clades, whereas *dfrA* was predominantly associated with the green clades. The bold sequences of the green clade represent one of the structures described in Figure S3. Red circles at nodes indicate bootstrap support values ≥80.

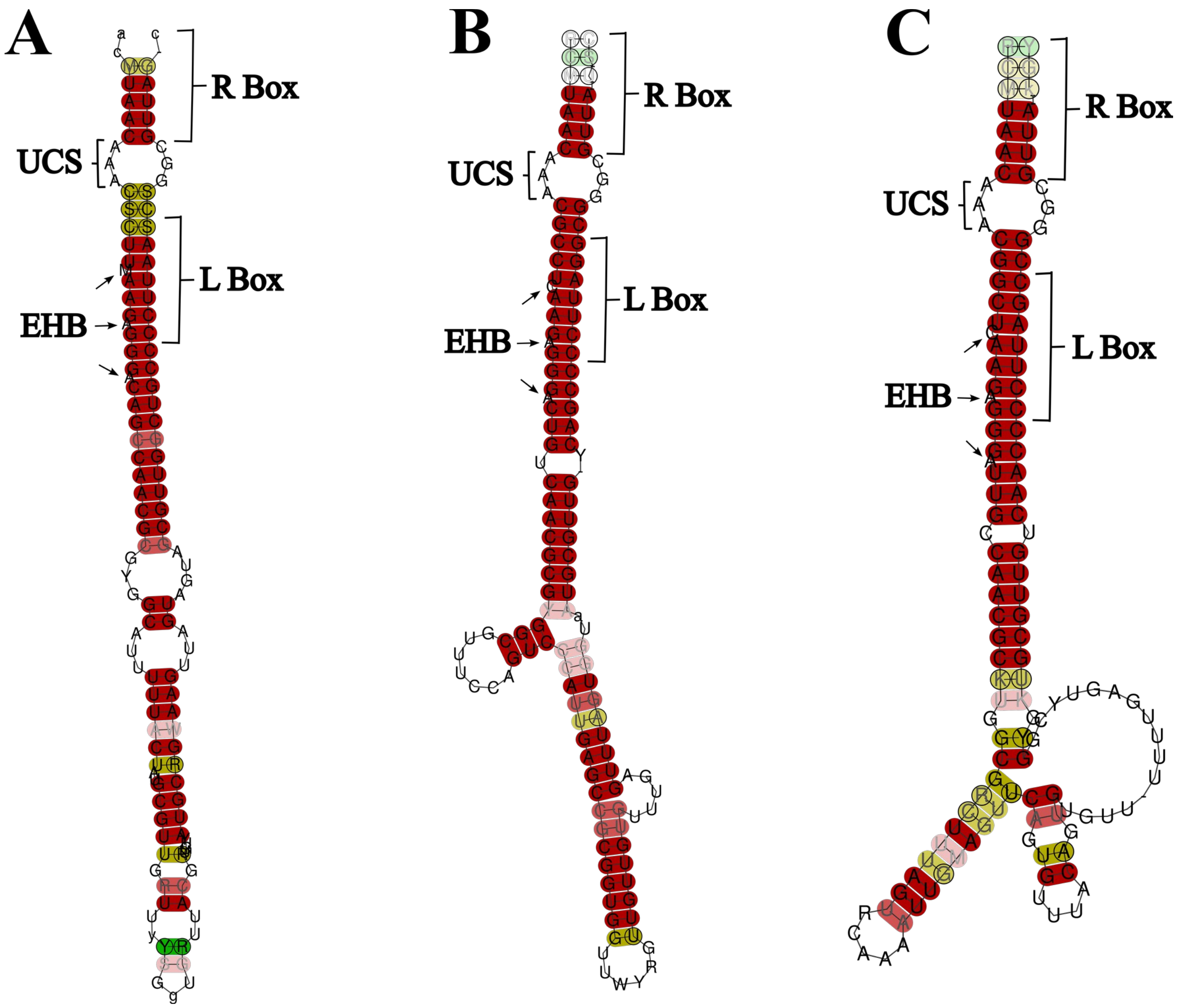

Figure S3. Predicted secondary structures of representative VXR sequences. Structures representing the predicted consensus secondary structures of (A) a *dfrA*-associated VXR cluster (ΔG = −62.94 kcal/mol; (B) the eight most prevalent VXR sequences in the dataset (ΔG = −58.38 kcal/mol; (C) and the seven most prevalent VXR sequences among non-*V. cholerae* genomes (ΔG = −58.23 kcal/mol. Structures were predicted using mLocARNA with the Mathews 2004 DNA thermodynamic parameters (dna_mathews2004.par; ViennaRNA Package). Colors indicate conservation of base-pair types in the consensus structure. Red denotes a single conserved base-pair type, whereas yellow and green indicate two and three different base-pair types, respectively. Color saturation reflects the consistency of the base pair across the sequences. The representative VXRs display a conserved stem–loop architecture despite sequence variation, consistent with structural conservation of these *attC*-associated repeats. EHB, extra helical bases (indicated by the arrows); UCS, unpaired central spacer.
